# Ambient Oxygen Promotes Tumorigenesis

**DOI:** 10.1371/journal.pone.0019785

**Published:** 2011-05-12

**Authors:** Ho Joong Sung, Wenzhe Ma, Matthew F. Starost, Cory U. Lago, Philip K. Lim, Michael N. Sack, Ju-Gyeong Kang, Ping-yuan Wang, Paul M. Hwang

**Affiliations:** 1 Center for Molecular Medicine, National Heart, Lung, and Blood Institute, National Institutes of Health, Bethesda, Maryland, United States of America; 2 Department of Biomedical Laboratory Science, College of Health Science, Eulji University, GyeongGi-Do, Korea; Dana-Farber Cancer Institute, United States of America

## Abstract

Oxygen serves as an essential factor for oxidative stress, and it has been shown to be a mutagen in bacteria. While it is well established that ambient oxygen can also cause genomic instability in cultured mammalian cells, its effect on *de novo* tumorigenesis at the organismal level is unclear. Herein, by decreasing ambient oxygen exposure, we report a ∼50% increase in the median tumor-free survival time of *p53−/−* mice. In the thymus, reducing oxygen exposure decreased the levels of oxidative DNA damage and RAG recombinase, both of which are known to promote lymphomagenesis in *p53−/−* mice. Oxygen is further shown to be associated with genomic instability in two additional cancer models involving the *APC* tumor suppressor gene and chemical carcinogenesis. Together, these observations represent the first report directly testing the effect of ambient oxygen on *de novo* tumorigenesis and provide important physiologic evidence demonstrating its critical role in increasing genomic instability *in vivo*.

## Introduction

Oxygen is essential for aerobic life and its deficiency is associated with disease. However, the dual nature of oxygen was recently demonstrated using a cell model with disruption of a gene critical for mitochondrial respiration [1]. While oxygen is required for oxidative phosphorylation to generate ATP, it also serves as the essential substrate for the generation of reactive oxygen species (ROS) implicated in tumorigenesis. Conceptually, oxygen toxicity, not bioenergetic efficiency, was initially proposed as the evolutionary driving force in the symbiotic theory of the mitochondrion [2]. Non-respiring cells have increased oxygen-associated genomic DNA damage demonstrating that the disposition of intracellular oxygen by the mitochondria plays an important role in maintaining genomic stability [1]. Previous studies have also suggested the important role of mitochondrial respiration in preventing oxidative stress and decreasing the incidence of cancer and other age-associated degenerative diseases [3], [4], [5], [6], [7].

As the essential substrate for oxidative stress, ambient oxygen could play an important role in DNA damage and cellular senescence. Indeed, lowering the oxygen exposure of cultured cells has been shown to decrease oxidative DNA damage and extend cellular lifespan [1], [8], [9]. Physiological levels of oxygen have been shown to be mutagenic in bacteria [10] and suggested to play an important role in promoting renal cancer formation *in vivo* [11], but whether ambient oxygen can directly affect *de novo* tumorigenesis has not been demonstrated. Although experimental data in this area remains sparse, epidemiologic analysis, adjusted for confounding factors such as natural radiation, shows decreased cancer incidence in human populations living at high altitude [12]. Such potentially important observations are difficult to interpret in the absence of a specific mechanism because of the various physiologic and genetic adaptations that can occur in humans living in low oxygen environments [13], [14]. Therefore, we decided to test the effect of reducing ambient oxygen exposure on *de novo* tumorigenesis and *in vivo* genomic instability, an important driver in pathogenesis, using three well-defined cancer models. These current observations serve as a first step to delineating the mechanisms that may underlie the association between oxygen exposure and cancer.

## Materials and Methods

### Ethics statement

Mice were bred and maintained in the approved animal housing facility of the National Institutes of Health, and all animal experiments were in accordance with the guidelines of the NIH animal care and use committee. The NIH ACUC approval identification number for this animal protocol is H-0126.

### Antibodies and reagents

Antibodies used in the study are as follows: Rabbit polyclonal RAG1 (K-20, Santa Cruz Biotechnology); monoclonal phospho-S-139 histone H2AX (γ-H2AX, clone JBW301, Millipore); monoclonal β-actin (AC-15, Sigma); rabbit monoclonal β-catenin (6B3) and cyclin D1 (DCS6, Cell Signaling). Chemicals used in the study are as follows: avidin-FITC; 7,12-dimethylbenz[a]anthracene (DMBA); and phorbol 12-myristate 13-acetate (TPA) (all from Sigma).

### Cell culture

The *p53+/+* and *p53−/−* HCT116 human colon cancer cell lines were provided by Bert Vogelstein, Johns Hopkins University, and grown in McCoy's 5A medium supplemented with 10% fetal bovine serum containing penicillin/streptomycin [15].

### Hypoxia chamber

A large hypoxia chamber (Coy Laboratory Product Inc.) capable of accommodating multiple standard-sized mouse cages with a live animal waste removal system was set at 10% O_2_, 0–0.5% CO_2_, 30–70% humidity at room temperature. These parameters were continuously monitored with internal and external probes through a tele-alarm service (Rees Scientific). All animal studies including endpoint determinations were performed in accordance with the guidelines and approval of the NIH animal care and use committee. In all animals used for tumor-free survival studies, necropsies were performed and histopathologic diagnoses confirmed by certified NIH veterinary pathologists.

### *p53−/−* mouse cancer model

C57BL/6J background strain wild-type and *p53−/−* mice (B6.129S2-*Trp53^tm1Tyj^*/J) were obtained from Jackson Laboratory and bred to obtain littermates. Mice were weaned in 21% oxygen (normoxia, room air) prior to randomization to 10% (hypoxia) or 21% oxygen according to animal care guidelines. Mice were closely monitored for any evidence of tumor or health deterioration. Animals were euthanized and necropsies performed when any external mass exceeded 2 cm in its largest dimension or when the mice became moribund.

### *APC^Min/+^* mouse polyposis model

Five week-old male *APC* ^Min/+^ mice (C57BL/6J-ApcMin/J) were obtained from Jackson Laboratory and maintained in 10% or 21% oxygen for 14 wk prior to sacrifice. The lumen of the small intestine was examined under a low power dissecting scope and the polyp diameters were measured using a caliper [16].

### Chemical skin carcinogenesis

Five-wk old female FVB mice (FVB/NJ, Jackson Laboratory) were used for the two-stage chemical skin carcinogenesis model as previously described [17]. DMBA at a dosage of 5 µg or 100 µg in 200 µl of acetone was applied to the shaved back of the mice followed 2 wk later by 12.5 µg TPA (twice a week) for 13 wk or 11 wk, respectively, prior to papilloma quantification. For the tumor endpoint survival study, TPA was applied until the defined endpoint of papilloma size(s) exceeding 2 cm in any dimension, ulceration or rupture of tumor, moribound state, or death.

### Blood oxygen saturation measurement

Arterial blood oxygen saturation was determined using a pulse oximetry sensor (MouseOx™ Oxymeter, STARR Life Sciences Corp.) attached to the tail of 10% oxygen acclimated mice. Oxygen saturation was converted to partial pressure (mm Hg) as previously reported [18].

### Physiological Measurements and Body Mass Composition

Blood samples were collected in EDTA anti-coagulant tubes for hematocrit measurements. The Bruker Minispec NMR analyzer (Bruker Optics) was used to measure body composition of non-anesthetized mice after up to 16 wk of 10% oxygen exposure (∼21 wk old) [19]. Average daily food intake was estimated by weighing the standard mouse chow in each cage over at least 4 wk.

### Xenograft tumor growth assay

Athymic nude mice (B6.Cg-Foxn1nu/J, Jackson Laboratory) were subcutaneously injected with 10×10^6^ cells into each hind limb and housed in the indicated oxygen condition. Tumor dimensions were measured using a digital caliper two times a week and volumes estimated as previously described (volume = (width^2^×length)/2) [20].

### Apoptosis Detection

Apoptotic cells were stained by the terminal deoxynucleotidyl transferase-mediated dUTP nick-end labeling (TUNEL) technique using paraffin-embedded tissue sections (Histoserve, Inc.). Images were captured using an Axioskop2 Plus fluorescence microscope with Axiovision software (version 4.6) and apoptotic nuclei were quantified.

### Blood GSH and GSSG measurement

Blood from wild-type C57BL/6J mice in room air or acclimated to 10% oxygen for at least 4 wk was collected in EDTA tubes and used for GSH and GSSG measurement according to the manufacturer's protocol (BIOXYTECH® GSH/GSSG-412 kit, OxiResearch). Absorbance signals were collected using the Victor3 plate reader (PerkinElmer).

### ROS measurement

Samples obtained from mice exposed to different oxygen concentrations were handled as rapidly as possible. The thymus tissue was gently dissociated into cell suspension (PBS) using sequential filtration through a nylon mesh (Gelman Laboratory) and a 40 µm cell strainer (BD Falcon) and further washed with PBS to remove tissue debris. The isolated thymocytes were counted, stained with 5 µM CM-H_2_DCFDA (Invitrogen) for 15 min at 37°C, and washed with PBS. DCF signal was collected and analyzed using FACSCalibur and CELLQusest™ software (version 3.3, BD Biosciences).

### 8-oxoG visualization with avidin-FITC

Frozen tissue sections (10 µm) were fixed in 2% paraformaldehyde PBS, permeabilized in 0.2% Triton X-100 PBS, RNA eliminated by RNase A (10 µg/ml) treatment for 1 hr at room temperature, and incubated in avidin-FITC (∼1∶200, Sigma) as previously described [21], [22]. Three PBS washes (5 min each) were performed between all steps. Vectashield mounting medium with DAPI (Vector Laboratories) was used to coverslip the slides and their images captured using an Axioskop2 Plus fluorescence microscope with Axiovision software (version 4.6).

### Genomic DNA 8-oxoG content measurement

Genomic DNA from tissues was isolated using the DNeasy kit (Qiagen) and 8-oxoG content determined using an ELISA kit according to manufacturer's protocol (8-OHdG Check Ultrasensitive ELISA, Multispecies Specificity, BioVendor). Absorbance signals were collected using the Victor3 plate reader (PerkinElmer).

### Telomere length measurement

Telomere length using 10 ng of purified genomic DNA was determined by a PCR-based technique as previously described [23]. Ct values of the telomeric repeats and of the single-copy *acidic ribosomal phosphoprotein PO* (*36B4*) gene were determined using SYBR Green real-time PCR on a 7900HT Sequence Detection System (Applied Biosystems) [23], [24]. Relative telomere length was determined by the ratio of telomere to 36B4 Ct values.

Telomere primers:
  F: 5′ CGG TTT GTT TGG GTT TGG GTT TGG GTT TGG GTT TGG GTT 3′
  R: 5′ GGC TTG CCT TAC CCT TAC CCT TAC CCT TAC CCT TAC CCT 3′
*36B4* primers:
  F: 5′ ACT GGT CTA GGA CCC GAG AAG 3′
  R: 5′ TCA ATG GTG CCT CTG GAG ATT 3′

### Western blotting

Tissues were homogenized in cold RIPA protein lysis buffer with protease inhibitor cocktail and frozen on dry ice for storage. Protein samples were heated for 5 min at 95°C prior to resolving and transferring in Novex Tris-Glycine gel and XCell II™ blot module (Invitrogen), respectively. Membranes were incubated overnight with primary antibody at 4°C and developed with HRP-conjugated secondary antibody (∼1∶10,000, Jackson Laboratory) using standard protocols.

### Quantification of HIF1-α activity by target mRNA expression

mRNA levels of the HIF1-α target genes *VEGF* and *LDHA* were measured by RT-PCR and normalized to a housekeeping gene *TIF* mRNA as previously described [24].

VEGF primers
  F: 5′-GTACTTGCAGATGTGACAAGCC-3′
  R: 5′-GGGTGTGTCTACAGGAATCC-3′
LDHA primers
  F: 5′- TGGCAGACTTGGCTGAGAGC-3′
  R: 5′- GGTGTGGTCTGCCTAGAAGC-3′

### Statistical analysis

*P* values were calculated using Student's *t*-test as indicated. Kaplan-Meier survival analyses were performed using the Prism 4 software (GraphPad Software, Inc.).

## Results

### Ambient oxygen increases DNA damage and promotes tumorigenesis in *p53−/−* mice

**Figure 1:**
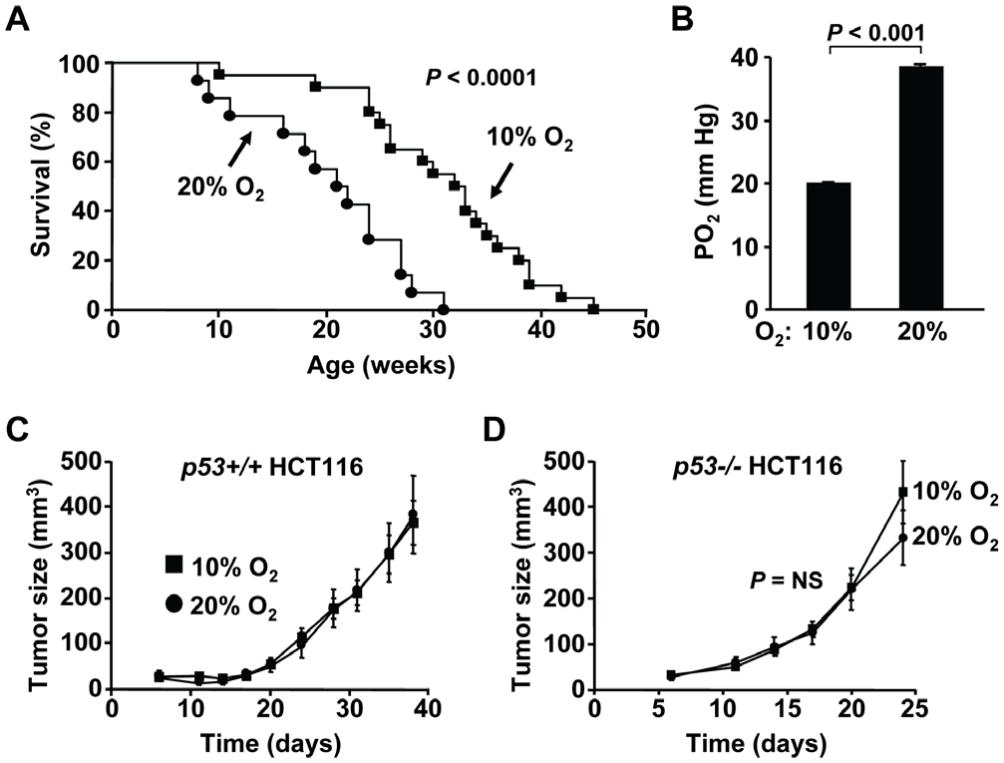
Ambient oxygen promotes tumorigenesis in *p53−/−* mice. **A.** *p53−/−* mice show increased median tumor-free survival time in 10% compared to 21% ambient oxygen by Kaplan-Meier survival analysis. After weaning in 21% oxygen (normoxia, room air), the *p53−/−* mice either remained in normoxia or were transferred to standard-sized mouse cages in a large 10% oxygen (hypoxia) chamber. Tumor-free survival period was defined as time to death with tumor diagnosis at necropsy or to an external tumor exceeding 2 cm in any dimension per institutional animal care and use committee guidelines (10% oxygen, n = 20; 21% oxygen, n = 14). **B.** Arterial oxygen saturation is proportional to ambient oxygen after acclimation indicating absence of significant supply-demand mismatch (partial pressure of oxygen, PO_2_). Data are shown as mean ± SEM, with n = 5. **C.** Decreased ambient oxygen does not inhibit the growth of *p53+/+* HCT116 cancer cell as xenografts. 10×10^6^ cells were injected into the contra-lateral hind limbs of athymic nude mice and immediately placed in their respective ambient oxygen conditions. Xenograft tumor growth was monitored over the time as shown (n = 10 each). **D.** Decreased ambient oxygen does not inhibit the growth of *p53−/−* HCT116 cancer cell as xenografts (n = 10 each).

To test the concept of ambient oxygen as a tumor promoter, we placed tumor-prone p53 deficient (*p53−/−*) mice with defective DNA damage response in free-living chronic hypoxia chambers (10% oxygen, equivalent to ∼5,000 meters above sea level). All animals were housed in normoxic conditions (room air, ∼21% oxygen) until weaning, at which time some remained in normoxia and others were transferred to hypoxia. The decreased oxygen exposure, even after earlier life in normoxia, resulted in a ∼50% longer median tumor-free survival time compared to animals that remained in normoxia (33 versus 22 weeks, respectively, *P*<0.0001) (Figure 1A). This survival data provided physiologic evidence that ambient oxygen can indeed promote tumorigenesis in a setting of reduced genomic protection such as found in *p53−/−* mice.

We first confirmed that tissue oxygenation is indeed decreased by hypoxia but that it is adequate to supply general metabolic needs. Notably, the partial pressure of oxygen in arterial blood was reduced by 50% as would be predicted from the magnitude of reduction in ambient oxygen (from 21% to 10%) while the hematocrit level was increased confirming chronic adaptation to hypoxia (Figure 1B and Figure S1) [25]. The correspondence between the relative reduction in arterial blood oxygenation and ambient oxygen level was consistent with previously published direct measurements of tissue oxygen concentration in animals [25], and it also suggested that the adaptive changes were sufficient to meet the metabolic requirements of mice in 10% oxygen following acclimation. Furthermore, no significant physiological changes were noted between the 10% and 21% oxygen conditions, as assessed by body weight, body mass composition and food intake, indicating that the animals were in overall good health before cancer development (Figure S2).

We next sought to provide an explanation for the improved tumor-free survival under the mildly low oxygen condition. It is formally possible that the decreased oxygen concentration in the hypoxia chamber could bioenergetically limit the growth of cancer cells by decreasing oxidative phosphorylation. To test this possibility, we examined the growth rate of the established human colon cancer HCT116 cells as xenografts in 10% versus 21% oxygen. The xenograft growth rates were similar indicating that the *in vivo* proliferation of established cancer cells is not inhibited by a lower ambient oxygen level and thus does not serve to explain the increased tumor-free survival in hypoxia (Figure 1C). As the p53 deficient state may influence tumor growth in hypoxia, we also measured the xenograft growth of *p53−/−* HCT116 cells. As previously observed, *p53−/−* cells appeared to grow more rapidly compared to isogenic *p53+/+* cells [15], but their growth rates were also not affected by the tested ambient oxygen levels (Figure 1D).

During the course of our study, the majority of *p53−/−* mice in 21% oxygen developed thymic lymphomas as previously described [26]. No qualitative differences could be detected in the incidence of lymphoma that developed under the different oxygen conditions as assessed by histological appearance, presence of apoptotic nuclei, or metastasis (Figure S3A and S3B). However, it was notable that in the 10% versus 21% oxygen condition, there was a decreased trend of lymphoma development and an increased trend for non-lymphoma tumors which were histologically confirmed to ensure that they were not metastatic thymic lymphomas (Figure S3C). One explanation for this phenomenon could be that lowering oxygen exposure delays lymphomagenesis and that the subsequent increase in lifespan allows for the emergence of other types of tumors that otherwise would not have been observed. In support of this notion, most of the non-lymphoma tumors (78%) occurred in mice that survived beyond 6 months, the typical lifespan of *p53−/−* mice in normoxia.

Our current observation of increased tumor-free survival under reduced ambient oxygen in *p53−/−* mice appeared to be inconsistent with the observation that hypoxia, through HIF1-α for example, may promote the growth of cancer cells [27]. This suggested that we were observing a fundamentally different aspect of tumorigenesis compared to the studies using established cancer cell lines by *in vitro* or xenografts assays which reflect the proliferation of cancer cells. Because ambient oxygen can cause oxidative DNA damage *in vitro* [1], it could also initiate tumorigenesis through an increase in genomic instability *in vivo*. Thus, we hypothesized that reduced oxygen exposure might result in lower levels of oxidative stress and genomic DNA damage in a tumor prone tissue, such as the thymus, with a subsequent delay in lymphomagenesis.

**Figure 2:**
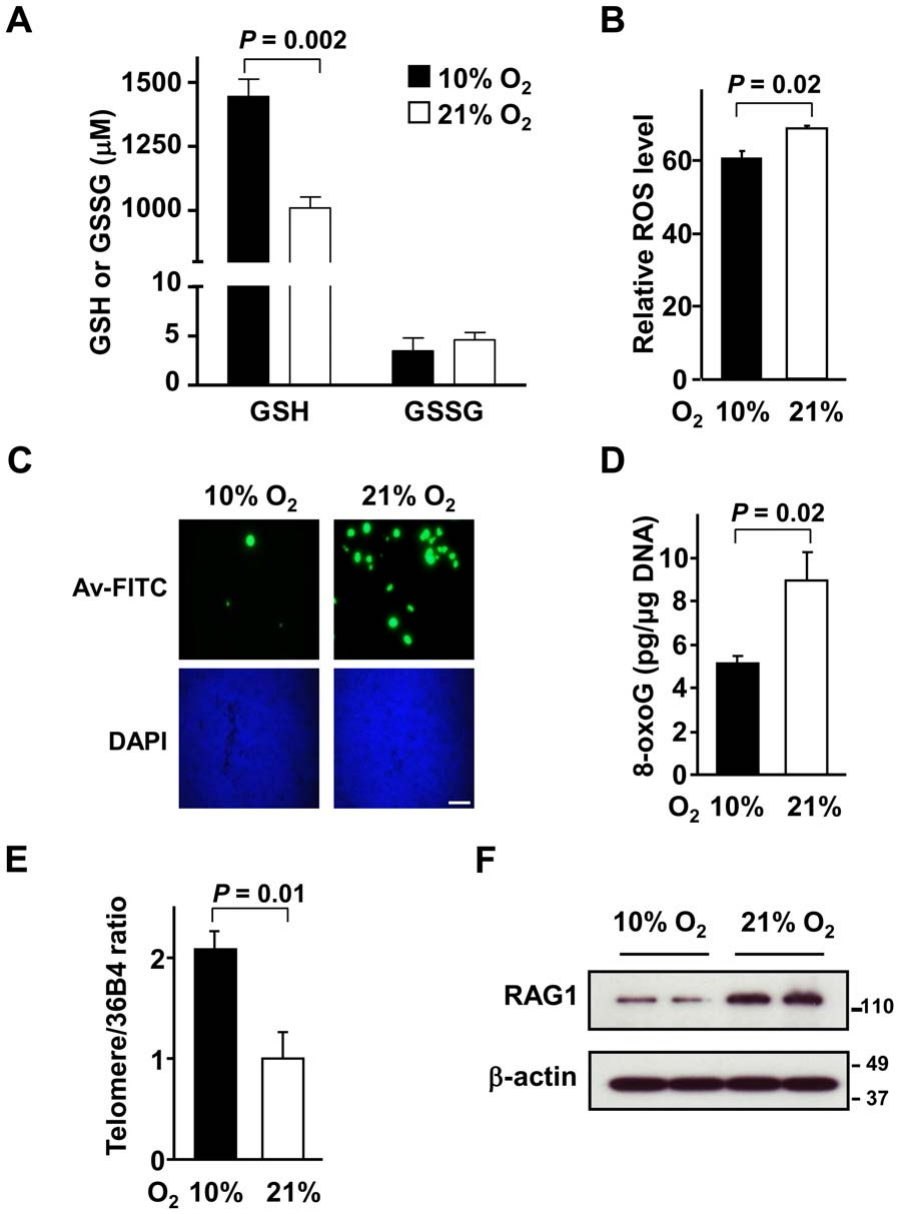
Lowering oxygen exposure reduces oxidative stress, DNA damage and genomic instability in thymus. **A.** Changes in reduced (GSH) and oxidized (GSSG) glutathione levels show increased antioxidant capacity in blood of mice after chronic adaptation to the 10% oxygen condition. Data shown as mean ± SEM, with n = 4. **B.** Decreased ROS levels measured by DCF FACS in cells isolated from the thymus of mice in 10% oxygen. Data shown as mean ± SEM, with n = 5. **C.** Representative images of decreased oxidative DNA damage detected by avidin-FITC (Av-FITC) staining for 8-oxoG in thymus tissue from *p53−/−* mouse exposed to 10% versus 21% oxygen for 2 to 4 wk. Nuclei counterstaining with DAPI show similar densities in the tissue. Scale bar, 100 µm (originally 63× magnification). **D.** Decreased oxidative DNA damage quantified by 8-oxoG enzyme-linked immunosorbent assay (ELISA) in thymus tissue from *p53−/−* mice exposed to 10% compared to 21% oxygen. Absolute value of 8-oxoG (pg/µg genomic DNA) shown as mean ± SEM, with n = 3. **E.** Increased relative telomere length measured by RT-PCR of genomic DNA from thymus tissue of *p53−/−* mice exposed to 10% versus 21% oxygen. Data shown as mean ± SEM, with n = 3. **F.** Decreased RAG1 protein level measured by western blotting in 10% versus 21% oxygen. Samples shown are from two separate animals in each oxygen condition and β-actin serves as protein loading control.

Glutathione is the major free radical scavenger and an important marker of oxidative stress. To show that mice exposed to a lower ambient oxygen level had less oxidative stress, we measured both reduced (GSH) and oxidized (GSSG) glutathione in blood of mice chronically adapted to 10% oxygen. The GSH concentration increased by 43% in the 10% oxygen condition compared to room air while there was no significant difference in GSSG levels within the detection limits of the assay (Figure 2A). This increase in GSH, which is mostly contained in erythrocytes [28], could not be fully explained by the 23% increase in hematocrit after hypoxic adaptation (Figure S1), suggesting that physiologic hypoxia reduces oxidative stress and increases antioxidant capacity. Furthermore, we observed a decrease in ROS levels by DCF staining of cells dissociated from the thymus of mice chronically adapted to 10% versus 21% oxygen (Figure 2B). Consistent with these findings of reduced oxidative stress under relatively mild hypoxia, we observed a significant reduction in avidin-FITC staining, which detects oxidatively modified DNA base 8-oxoguanine (8-oxoG), in the thymus of *p53−/−* mice chronically adapted to hypoxia prior to the overt development of lymphoma (Figure 2C). The quantification of 8-oxoG by immunoassay using genomic DNA prepared from thymus tissue confirmed the increase in oxidative DNA damage visualized by avidin-FITC in 21% oxygen (Figure 2C and 2D).

Oxidative stress has also been associated with telomere shortening [29] and dysfunctional telomerase has been linked to the increased incidence of cancer in *p53−/−* mice [30]. Conversely, the low oxygen niche of hematopoietic stem cells has been associated with increased telomerase activity [31]. Concordant with these observations, the genomic DNA of thymus tissue showed significant oxygen-associated shortening of telomere length as another marker of DNA damage and genomic instability (Figure 2E). Furthermore, a recent study has revealed that blocking T cells at stages of high recombination-activating gene (RAG) activity accelerates lymphomagenesis in *p53−/−* mice [32] while the absence of *RAG1* significantly increases the latency of lymphomagenesis in p53 inactivated states [33]. Therefore, we examined whether oxygen might influence RAG expression in the thymus. In 10% oxygen, there was a significant decrease in the level of RAG1 protein expression compared to 21% oxygen. Together, these observations suggested that oxygen can cause genomic instability through more than one mechanism such as that involving RAG, which is known to cause DNA double-strand breaks in the setting of deficient DNA damage response (Figure 2F) [32]. Although we have not shown that one specific pathway causally explains our tumor-free survival data, the observation of decreased genomic instability under the low oxygen condition using three different markers strongly suggests its involvement in this phenomenon.

### Ambient oxygen increases genomic instability at the APC locus and promotes polyp formation in *APC^Min/+^* mice

**Figure 3:**
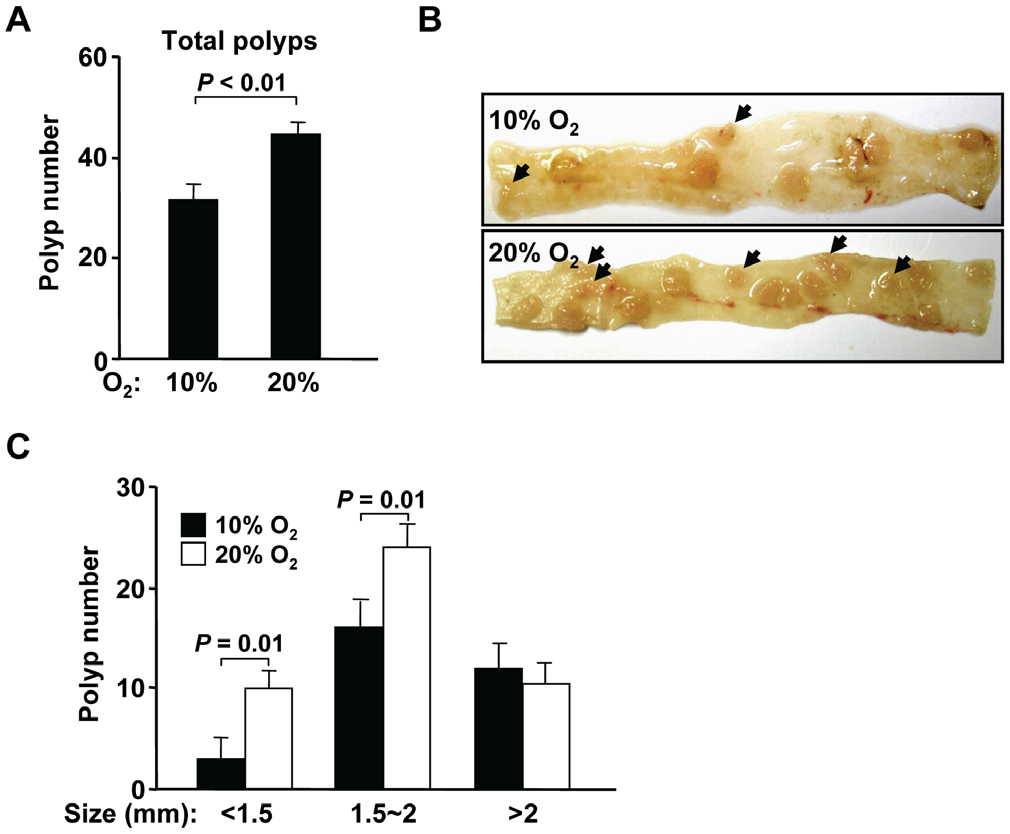
Ambient oxygen promotes tumorigenesis in *APC^Min/+^* mice. **A.** Significant decrease in the total number of small intestinal polyps per mouse in 19 wk old *APC^Min/+^* mice that were placed in 10% versus 21% oxygen for 14 wk, starting at 5 wk of age. Data shown as mean ± SEM, with n = 12. **B.** Decreased smaller diameter polyps (black arrowheads) in representative intestinal segments from mice in 10% compared to 21% oxygen. **C.** Quantification of polyps classified by size (polyp diameter, mm) in 10% versus 21% oxygen. The relative reduction in polyp number in 10% versus 21% oxygen is greatest for the smallest diameter polyps (<1.5 mm). Data shown as mean ± SEM, with n = 12.

To further expand on our initial observation that oxygen promotes genomic instability and tumorigenesis, we examined the effect of ambient oxygen on the *APC^Min/+^* mouse model of intestinal neoplasia wherein the inactivation of the wild-type *APC* allele initiates the formation of polyps [34], [35]. Thus, polyp formation serves as a functional readout of genomic instability. Five weeks after birth, *APC^Min/+^* mice were placed in 10% oxygen and their intestines assessed for polyp formation after 14 wk (age ∼19 wk). We observed a significant reduction in the total number of intestinal polyps under lower oxygen compared to the room air condition (Figure 3A). When the size distribution of the intestinal polyps was examined, the number of smaller polyps appeared to be more significantly affected by changes in oxygen exposure (Figure 3B). The relative difference in the number of polyps in 10% versus 21% ambient oxygen was greater for smaller diameter polyps (<1.5 mm and 1.5∼2 mm) while there was no significant difference in larger polyps (>2 mm) (Figure 3C). One interpretation of this data is that the larger, likely older, polyps were initiated sometime during the first 5 weeks of life when both groups of mice were exposed to the same room air condition and therefore were similar in number. In contrast, the smaller polyps, likely representing more recently initiated polyps after reduced oxygen exposure, displayed the largest relative difference (Figure 3C). Thus, this well-defined model served to functionally demonstrate that ambient oxygen is involved in promoting the polyp formation by affecting genomic stability at the wild-type *APC* gene locus.

**Figure 4:**
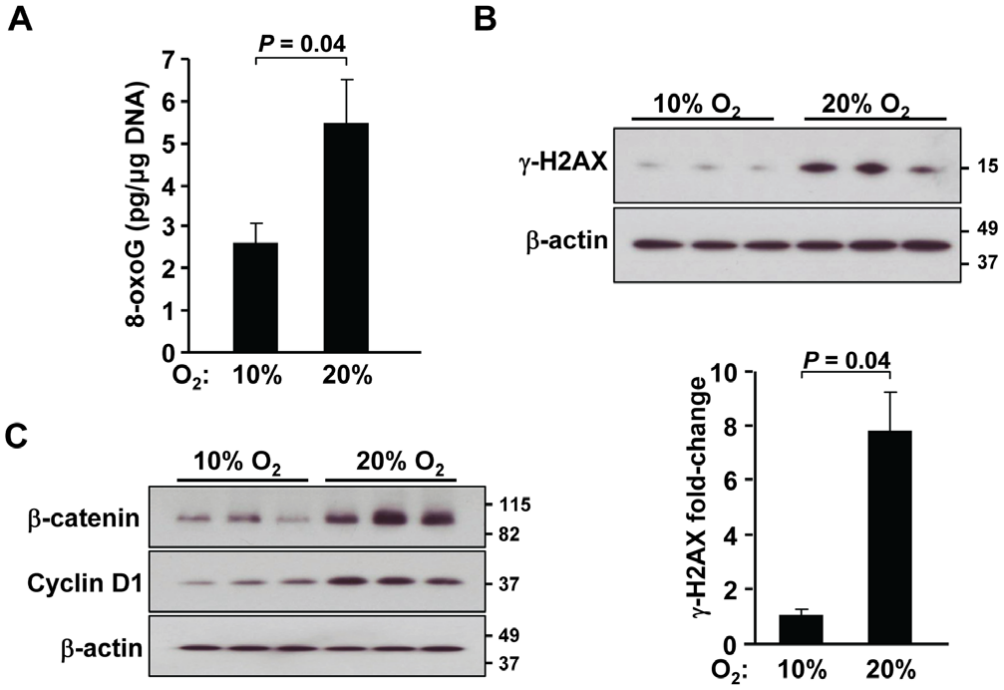
Lowering oxygen exposure prevents DNA damage and β-catenin accumulation in the intestine. **A.** Decreased oxidative DNA damage as measured by 8-oxoG (pg/µg genomic DNA) ELISA in polyp-free segments of intestine from 10% compared to 21% oxygen *APC^Min/+^* mice. Data shown as mean ± SEM, with n = 3. **B.** Decreased DNA damage response observed by γ-H2AX western blotting in polyp-free intestine of 10% compared to 21% oxygen *APC^Min/+^* mice. Relative intensity quantified by densitometric scanning of γ-H2AX normalized to β-actin protein (graph), n = 3. **C.** Decreased protein levels of β-catenin and its transcriptional target cyclin D1 in polyp-free intestine of 10% compared to 21% oxygen *APC^Min/+^* mice. Representative samples in each oxygen condition and β-actin serves as protein loading control, n = 3.

As with the *p53−/−* mouse thymus tissue, we also measured the levels of oxidatively modified genomic DNA in the small intestines of the *APC^Min/+^* mouse. Even in segments of intestine appearing normal, reduced ambient oxygen resulted in significantly lower 8-oxoG DNA levels that were paralleled by the decreased DNA damage response marker γ-H2AX (Figure 4A and 4B). As APC mediates the degradation of cytoplasmic β-catenin, reduced inactivation of the *APC* locus in 10% oxygen would be expected to result in decreased β-catenin levels along with its transcriptional target cyclin D1 that is involved in cell proliferation [36]. Indeed, tissue levels of both β-catenin and cyclin D1 protein were reduced in 10% oxygen, consistent with the decrease in polyp formation (Figure 4D).

### Ambient oxygen is involved in initiating chemical skin carcinogenesis

Hypoxia causes physiologic adaptations to improve tissue oxygenation such as the observed increase in hematocrit. However, the oxygen in the superficial layer of the skin is largely supplied by direct exposure to air so that tissue oxygenation can be more predictably altered by changing ambient oxygen conditions [37]. To minimize the effects of systemic adaptations to hypoxia, we used the two-stage chemical skin carcinogenesis model in which the topical application of the tumor initiating mutagen DMBA is followed by longer term treatment with phorbol esters (TPA).

**Figure 5:**
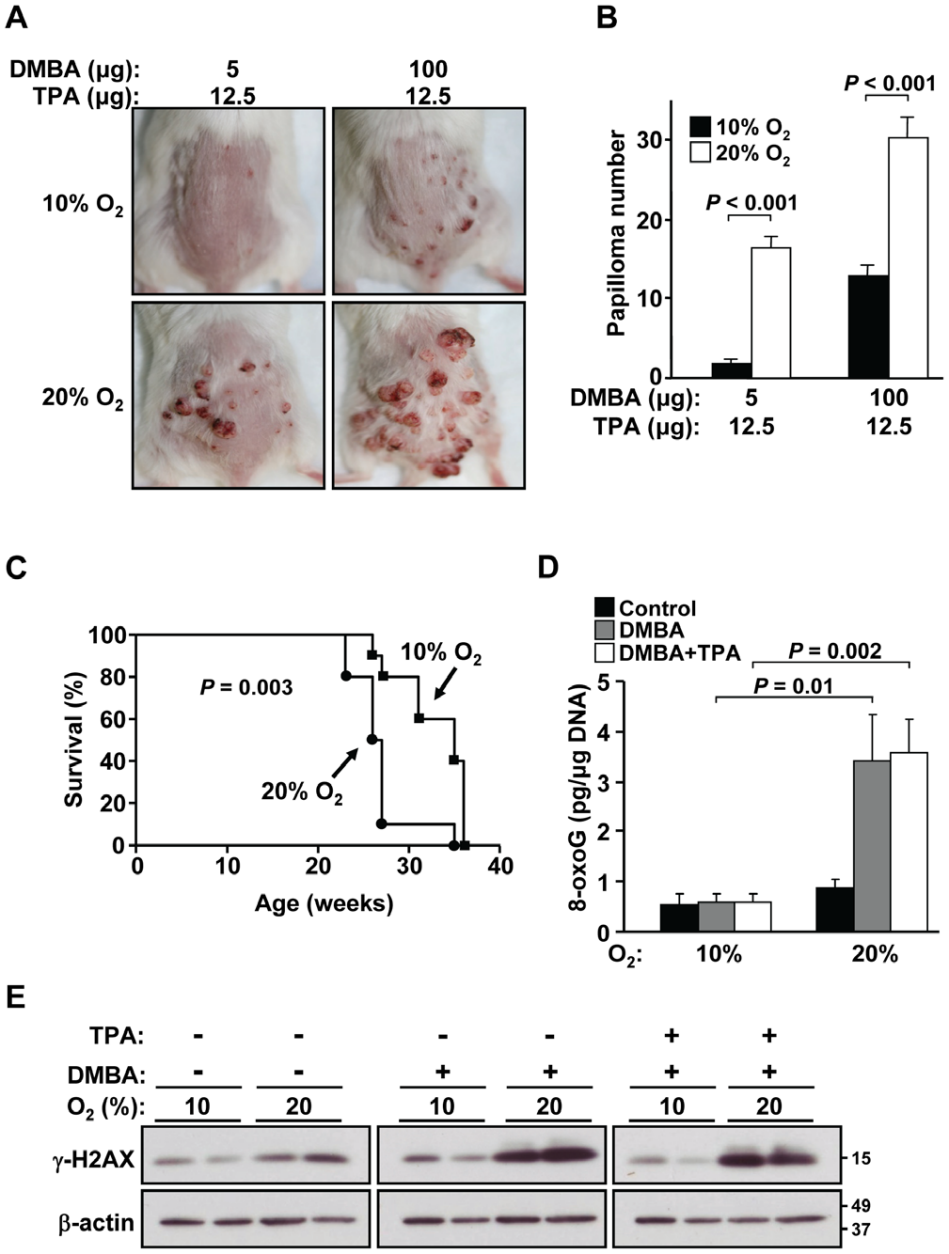
Ambient oxygen promotes chemical skin carcinogenesis. **A.** Decreased skin papilloma formation using the two-stage chemical skin carcinogenesis model in 10% compared to 21% ambient oxygen. Representative images are shown for standard (5 µg) or high (100 µg) topical dose of DMBA followed by chronic TPA application. **B.** Decreased papilloma number per mouse in 10% compared to 21% oxygen. Data shown as mean ± SEM, with n = 10 to 15. **C.** Increased median survival time to tumor endpoint in 10% compared to 21% oxygen by Kaplan-Meier survival analysis. The standard dose of DMBA followed by chronic TPA application was used until the defined tumor endpoint of papilloma size(s) exceeding 2 cm in any dimension, significant ulceration or rupture of tumor, moribound state, or death. 10% oxygen, n = 10; 21% oxygen, n = 10. **D.** Decreased oxidative DNA damage quantified by 8-oxoG ELISA in DMBA treated skin tissue of mice in 10% compared to 21% oxygen. DMBA (100 µg) and TPA (12.5 µg) were applied according to skin per protocol, animals were exposed to the indicated oxygen conditions, and skin tissue was harvested prior to the development of papillomas at 7 wk. Absolute value of 8-oxoG (pg/µg genomic DNA) shown as mean ± SEM, with n = 3. **E.** Confirmation of decreased DNA damage by protein levels of γ-H2AX in skin tissue from treated mice in 10% compared to 21% oxygen. β-actin serves as protein loading control and samples are shown from two separate animals for each condition as duplicates.

Under the 10% oxygen condition, there was a marked reduction in the formation of skin papillomas at both the standard (5 µg) and high (100 µg) dose of DMBA (Figure 5A and 5B). Using the standard DMBA dose, the median survival time to reach our predetermined endpoint (measured by skin tumor size and burden) was significantly delayed from 26 wk in 21% oxygen to 35 wk in 10% oxygen (*P* = 0.003, Figure 5C). This significant increase in median survival time using a cancer model that is different from the *p53−/−* mouse further confirmed the pro-tumorigenic effect of oxygen. Here, we also examined whether the oxidative modification of genomic DNA was affected by oxygen exposure in the skin. Compared to controls, DMBA caused a significant increase in the level of oxidatively modified genomic DNA that was highly dependent on 21% ambient oxygen (Figure 5D). The application of TPA did not alter the level of DNA damage, consistent with its proliferative and non-mutagenic mechanism of action. Furthermore, the levels of the DNA damage response protein γ-H2AX closely paralleled the 8-oxo-G measurements in the respective tissue samples (Figure 5E).

## Discussion

By reducing ambient oxygen exposure in three separate *de novo* cancer models, our study shows the potential importance of oxygen in influencing the initiation of tumorigenesis. Although large human epidemiologic datasets have correlated exposure to higher altitudes with lower cancer incidence, our study provides the first controlled demonstration that lower ambient oxygen levels delay tumorigenesis, independent of other variables such as barometric pressure and ultraviolet exposure associated with high altitude [12]. Our data also provide physiological evidence for the critical role of oxidative stress in increasing genomic instability, an essential driver in the multi-step process of tumorigenesis [38]. Most importantly, our study provides proof of concept that it is possible to alter the time course of tumor development by the simple modulation of ambient oxygen, the essential factor for oxidative stress.

We have previously shown that increased intracellular oxygen levels caused by the disruption of mitochondrial function may contribute to increased ROS generation and genomic DNA damage [1]. Thus, the current *in vivo* data support the *in vitro* observation that oxygen consumption via mitochondrial respiration may serve a fundamentally important function to guard against oxygen-associated genotoxicity. A previous study has reported that the antioxidant N-acetyl cysteine (NAC) can delay tumorigenesis in p53-deficient mice, underscoring the importance of p53's antioxidant activities in tumor suppression [22], [39]. Our study provides important complementary data to validate this prior observation which used a pharmacologic agent. Furthermore, the current study is unique in that through a physiological manipulation, we have demonstrated the importance of oxygen in modulating genomic instability.

Although our results show that lowering oxygen reduces genomic instability and tumorigenesis *in vivo*, hypoxic conditions in various systems have also been shown to promote tumor cell growth through HIF1-α induction [27], [40]. Conversely, established cancer cells engineered to over-express myoglobin, thus containing higher intracellular oxygen and lower HIF1-α levels, exhibit a decrease in xenograft tumor growth [41]. However, in our experiments tissues obtained from mice exposed to 10% oxygen did not show a significant increase in HIF1-α activity at this level of physiologically adaptable hypoxia (Figure S4). Thus, our observations are more likely to stem from a delay in tumor initiation due to reduced genomic instability in low oxygen rather than reflect the growth activities known to be stimulated by HIF1-α in established tumor cells.

Supraphysiologic levels of oxygen, including hyperbaric conditions, have been shown to inhibit tumorigenesis [42], [43]. However, oxidative stress responses such as p53 stabilization may contribute to inhibiting tumor growth [44], [45]. As demonstrated by many elegant studies, ROS plays multiple roles depending on various factors. ROS as a signaling molecule is necessary for cell proliferation [46], but at higher levels it can also induce cell death and prevent tumor growth [47]. Together with these observations, our results reveal the complexity of tumorigenesis and the dual nature of oxygen. Oxygen can promote cancer initiation by increasing genomic instability through oxidative stress while also having the potential to inhibit the growth of established tumor cells through specific signaling mechanisms [41].

The lessons from our current study may be applicable to human health. Supplemental oxygen is ubiquitously employed in clinical medicine because of its immediate benefits for energy production while the less apparent potential for genotoxicity can be neglected. Our work may provide a biological mechanism for important clinical observations such as the increased cancer risk of neonates exposed to supplemental oxygen or of babies conceived through *in vitro* fertilization, which may be performed under 21% oxygen whereas the oxygen concentration in the uterus is 5- to 10-fold lower [48], [49], [50], [51]. Although it is difficult to control for the many variables that are associated with the requirement for oxygen therapy, our experiments reveal the potential genotoxicity of oxygen which may be more immediately relevant to the clinics. Thus, minimizing oxygen exposure at early developmental stages such as in neonates or of *in vitro* fertilized oocytes prior to uterine implantation may potentially decrease the risk of cancer over a lifetime. Similarly, although antioxidants do not appear to have tumor suppressive effects in the general population [52], [53], targeting individuals who have specific inherited cancer susceptibility syndromes due to defects in DNA repair to strategies of reducing oxygen exposure or antioxidant therapy may yield marked benefits.

## Supporting Information

Figure S1 Blood hematocrit is increased after chronic adaptation to 10% oxygen. Data are shown as mean ± SEM, with n = 6 to 9.
(TIF)

Figure S2 No significant difference in body weight, body mass composition or food intake in the 10% versus 21% oxygen condition. **A.** Body weights of male and female *p53*−/− mice in 10% oxygen versus 21% oxygen are shown over time. Mice with evidence of tumor were excluded. Note that the body weights of the 10% oxygen group span a longer period due to longer tumor-free survival time (n = 4 to 17 in each group). **B.** Body mass composition (% muscle, fat or free fluid of total body weight) was measured in non-anesthetized mice that were housed in 10% or 21% oxygen for up to 16 wk. Data are shown as mean ± SEM, with n = 3. **C.** Average food intake per day (g/d) by each mouse measured over a 4 wk period. Data are shown as mean ± SEM, with n = 5.
(TIF)

Figure S3 Lymphoma characteristics of *p53−/−* mice in 10% versus 21% ambient oxygen. **A.** Representative images of TUNEL stained thymic lymphoma show no significant differences in apoptosis between 10% and 21% oxygen conditions. The arrowheads indicate positively stained cells. Right panel shows the quantification of apoptotic nuclei per high power field in 5 to 10 separate regions. Data are shown as mean ± SEM, with n = 3. Scale bar, 20 µm (originally 40× magnification). **B.** No significant difference in the fraction of mice with metastatic lymphoma as determined by necropsy (10% oxygen, n = 20; 21% oxygen, n = 14). **C.** There is a trend of increased incidence of non-lymphoma tumors in 10% compared to 21% ambient oxygen condition (10% oxygen, n = 20; 21% oxygen, n = 14).
(TIF)

Figure S4 Expression levels of HIF1-α target genes are not significantly increased in 10% versus 21% oxygen. The relative levels of VEGF and LDHA mRNA as markers of HIF1-α activity were measured in liver, spleen and thymus of *p53−/−* mice. Mice were acclimated to 10% oxygen for at least 2 to 4 wk prior to tissue harvest. Data are shown as mean ± SEM, with n = 3.
(TIF)

## References

[1] Sung HJ, Ma W, Wang P-y, Hynes J, O'Riordan TC (2010). Mitochondrial respiration protects against oxygen-associated DNA damage.. Nat Commun.

[2] Margulis L (1993). Symbiosis in Cell Evolution.

[3] Lagouge M, Argmann C, Gerhart-Hines Z, Meziane H, Lerin C (2006). Resveratrol improves mitochondrial function and protects against metabolic disease by activating SIRT1 and PGC-1alpha.. Cell.

[4] St-Pierre J, Drori S, Uldry M, Silvaggi JM, Rhee J (2006). Suppression of reactive oxygen species and neurodegeneration by the PGC-1 transcriptional coactivators.. Cell.

[5] Finkel T, Serrano M, Blasco MA (2007). The common biology of cancer and ageing.. Nature.

[6] Guarente L (2008). Mitochondria–a nexus for aging, calorie restriction, and sirtuins?. Cell.

[7] Liu J, Cao L, Chen J, Song S, Lee IH (2009). Bmi1 regulates mitochondrial function and the DNA damage response pathway.. Nature.

[8] Packer L, Fuehr K (1977). Low oxygen concentration extends the lifespan of cultured human diploid cells.. Nature.

[9] Parrinello S, Samper E, Krtolica A, Goldstein J, Melov S (2003). Oxygen sensitivity severely limits the replicative lifespan of murine fibroblasts.. Nat Cell Biol.

[10] Bruyninckx WJ, Mason HS, Morse SA (1978). Are physiological oxygen concentrations mutagenic?. Nature.

[11] Welford SM, Dorie MJ, Li X, Haase VH, Giaccia AJ (2010). Renal oxygenation suppresses VHL loss-induced senescence that is caused by increased sensitivity to oxidative stress.. Mol Cell Biol.

[12] Weinberg CR, Brown KG, Hoel DG (1987). Altitude, radiation, and mortality from cancer and heart disease.. Radiat Res.

[13] Simonson TS, Yang Y, Huff CD, Yun H, Qin G (2010). Genetic evidence for high-altitude adaptation in Tibet.. Science.

[14] Hochachka PW (1998). Mechanism and evolution of hypoxia-tolerance in humans.. J Exp Biol.

[15] Bunz F, Dutriaux A, Lengauer C, Waldman T, Zhou S (1998). Requirement for p53 and p21 to sustain G2 arrest after DNA damage.. Science.

[16] Cooper HS, Chang WC, Coudry R, Gary MA, Everley L (2005). Generation of a unique strain of multiple intestinal neoplasia (Apc(+/Min-FCCC)) mice with significantly increased numbers of colorectal adenomas.. Mol Carcinog.

[17] Amornphimoltham P, Leelahavanichkul K, Molinolo A, Patel V, Gutkind JS (2008). Inhibition of Mammalian target of rapamycin by rapamycin causes the regression of carcinogen-induced skin tumor lesions.. Clin Cancer Res.

[18] Uchida K, Reilly MP, Asakura T (1998). Molecular stability and function of mouse hemoglobins.. Zoological Science.

[19] Heron-Milhavet L, Haluzik M, Yakar S, Gavrilova O, Pack S (2004). Muscle-specific overexpression of CD36 reverses the insulin resistance and diabetes of MKR mice.. Endocrinology.

[20] Al-Ejeh F, Darby JM, Brown MP (2009). Chemotherapy synergizes with radioimmunotherapy targeting La autoantigen in tumors.. PLoS ONE.

[21] Neumann CA, Krause DS, Carman CV, Das S, Dubey DP (2003). Essential role for the peroxiredoxin Prdx1 in erythrocyte antioxidant defence and tumour suppression.. Nature.

[22] Sablina AA, Budanov AV, Ilyinskaya GV, Agapova LS, Kravchenko JE (2005). The antioxidant function of the p53 tumor suppressor.. Nat Med.

[23] Callicott RJ, Womack JE (2006). Real-time PCR assay for measurement of mouse telomeres.. Comp Med.

[24] Patino WD, Mian OY, Kang JG, Matoba S, Bartlett LD (2005). Circulating transcriptome reveals markers of atherosclerosis.. Proc Natl Acad Sci U S A.

[25] Braun RD, Lanzen JL, Snyder SA, Dewhirst MW (2001). Comparison of tumor and normal tissue oxygen tension measurements using OxyLite or microelectrodes in rodents.. Am J Physiol Heart Circ Physiol.

[26] Jacks T, Remington L, Williams BO, Schmitt EM, Halachmi S (1994). Tumor spectrum analysis in p53-mutant mice.. Curr Biol.

[27] Semenza GL (2010). Defining the role of hypoxia-inducible factor 1 in cancer biology and therapeutics.. Oncogene.

[28] Unt E, Kairane C, Vaher I, Zilmer K (2008). Red blood cell and whole blood glutathione redox status in endurance-trained men following a ski marathon.. Journal of Sports Science and Medicine.

[29] Passos JF, Saretzki G, Ahmed S, Nelson G, Richter T (2007). Mitochondrial dysfunction accounts for the stochastic heterogeneity in telomere-dependent senescence.. PLoS Biol.

[30] Maser RS, DePinho RA (2002). Connecting chromosomes, crisis, and cancer.. Science.

[31] Jang YY, Sharkis SJ (2007). A low level of reactive oxygen species selects for primitive hematopoietic stem cells that may reside in the low-oxygenic niche.. Blood.

[32] Haines BB, Ryu CJ, Chang S, Protopopov A, Luch A (2006). Block of T cell development in P53-deficient mice accelerates development of lymphomas with characteristic RAG-dependent cytogenetic alterations.. Cancer Cell.

[33] Liao MJ, Zhang XX, Hill R, Gao J, Qumsiyeh MB (1998). No requirement for V(D)J recombination in p53-deficient thymic lymphoma.. Mol Cell Biol.

[34] Luongo C, Moser AR, Gledhill S, Dove WF (1994). Loss of Apc+ in intestinal adenomas from Min mice.. Cancer Res.

[35] Su LK, Kinzler KW, Vogelstein B, Preisinger AC, Moser AR (1992). Multiple intestinal neoplasia caused by a mutation in the murine homolog of the APC gene.. Science.

[36] Shtutman M, Zhurinsky J, Simcha I, Albanese C, D'Amico M (1999). The cyclin D1 gene is a target of the beta-catenin/LEF-1 pathway.. Proc Natl Acad Sci U S A.

[37] Stucker M, Struk A, Altmeyer P, Herde M, Baumgartl H (2002). The cutaneous uptake of atmospheric oxygen contributes significantly to the oxygen supply of human dermis and epidermis.. J Physiol.

[38] Fearon ER, Vogelstein B (1990). A genetic model for colorectal tumorigenesis.. Cell.

[39] Olovnikov IA, Kravchenko JE, Chumakov PM (2009). Homeostatic functions of the p53 tumor suppressor: regulation of energy metabolism and antioxidant defense.. Semin Cancer Biol.

[40] Gao P, Zhang H, Dinavahi R, Li F, Xiang Y (2007). HIF-dependent antitumorigenic effect of antioxidants in vivo.. Cancer Cell.

[41] Galluzzo M, Pennacchietti S, Rosano S, Comoglio PM, Michieli P (2009). Prevention of hypoxia by myoglobin expression in human tumor cells promotes differentiation and inhibits metastasis.. J Clin Invest.

[42] Lindenschmidt RC, Tryka AF, Witschi HP (1986). Inhibition of mouse lung tumor development by hyperoxia.. Cancer Res.

[43] Raa A, Stansberg C, Steen VM, Bjerkvig R, Reed RK (2007). Hyperoxia retards growth and induces apoptosis and loss of glands and blood vessels in DMBA-induced rat mammary tumors.. BMC Cancer.

[44] Das KC, Dashnamoorthy R (2004). Hyperoxia activates the ATR-Chk1 pathway and phosphorylates p53 at multiple sites.. Am J Physiol Lung Cell Mol Physiol.

[45] Kulkarni A, Das KC (2008). Differential roles of ATR and ATM in p53, Chk1, and histone H2AX phosphorylation in response to hyperoxia: ATR-dependent ATM activation.. Am J Physiol Lung Cell Mol Physiol.

[46] Sundaresan M, Yu ZX, Ferrans VJ, Irani K, Finkel T (1995). Requirement for generation of H2O2 for platelet-derived growth factor signal transduction.. Science.

[47] Tu HC, Ren D, Wang GX, Chen DY, Westergard TD (2009). The p53-cathepsin axis cooperates with ROS to activate programmed necrotic death upon DNA damage.. Proc Natl Acad Sci U S A.

[48] Vaupel P, Kallinowski F, Okunieff P (1989). Blood flow, oxygen and nutrient supply, and metabolic microenvironment of human tumors: a review.. Cancer Res.

[49] Fischer B, Bavister BD (1993). Oxygen tension in the oviduct and uterus of rhesus monkeys, hamsters and rabbits.. J Reprod Fertil.

[50] Spector LG, Klebanoff MA, Feusner JH, Georgieff MK, Ross JA (2005). Childhood cancer following neonatal oxygen supplementation.. J Pediatr.

[51] Kallen B, Finnstrom O, Lindam A, Nilsson E, Nygren KG (2010). Cancer risk in children and young adults conceived by in vitro fertilization.. Pediatrics.

[52] Janne PA, Mayer RJ (2000). Chemoprevention of colorectal cancer.. N Engl J Med.

[53] Bardia A, Tleyjeh IM, Cerhan JR, Sood AK, Limburg PJ (2008). Efficacy of antioxidant supplementation in reducing primary cancer incidence and mortality: systematic review and meta-analysis.. Mayo Clin Proc.

