# Supplementary figures and images for: Ambient Oxygen Promotes Tumorigenesis
Source: PLoS One. 2011 May 12;6(5):e19785. doi: 10.1371/journal.pone.0019785 (PMC3093396; doi:10.1371/journal.pone.0019785)

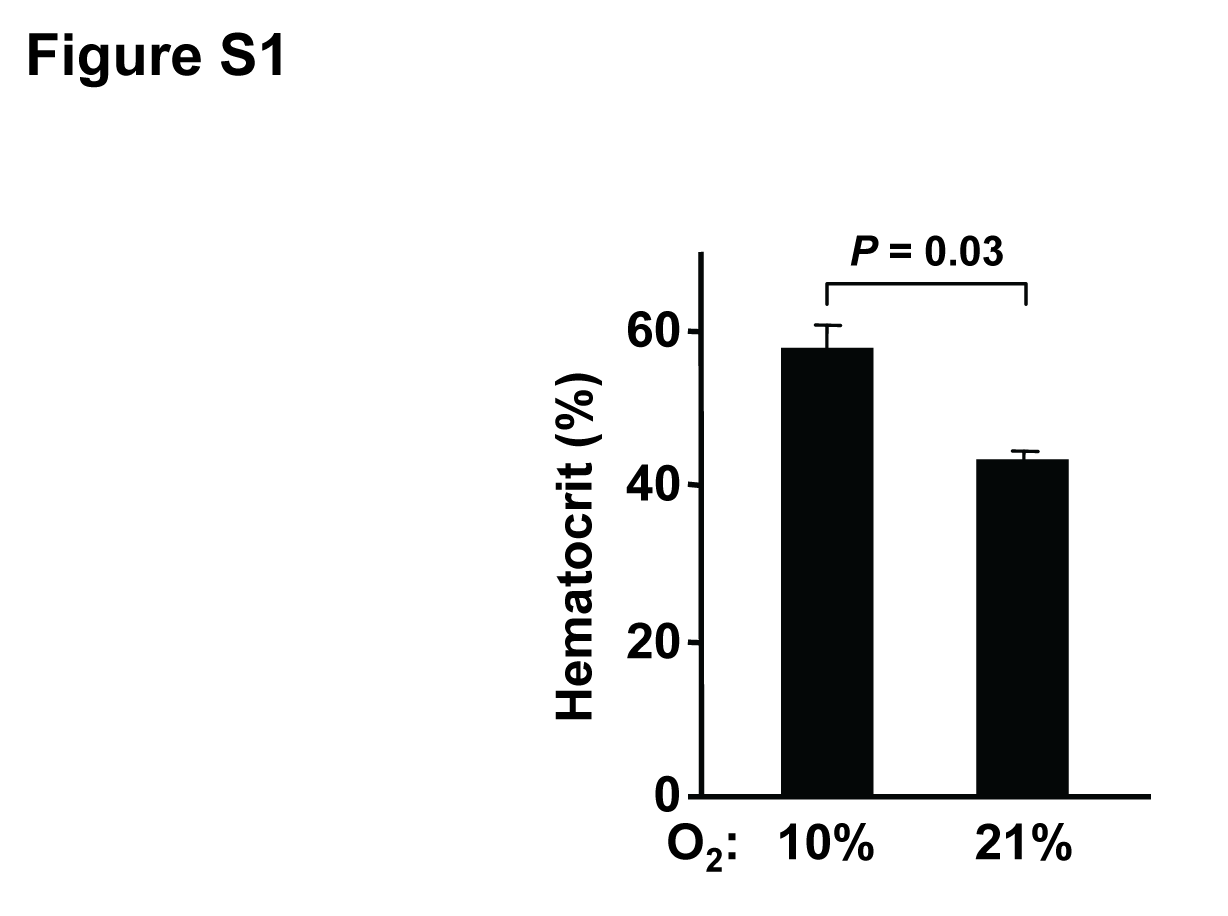

Supplement: Figure S1 — Blood hematocrit is increased after chronic adaptation to 10% oxygen. Data are shown as mean ± SEM, with n = 6 to 9. (TIF) [file pone.0019785.s001.tif]

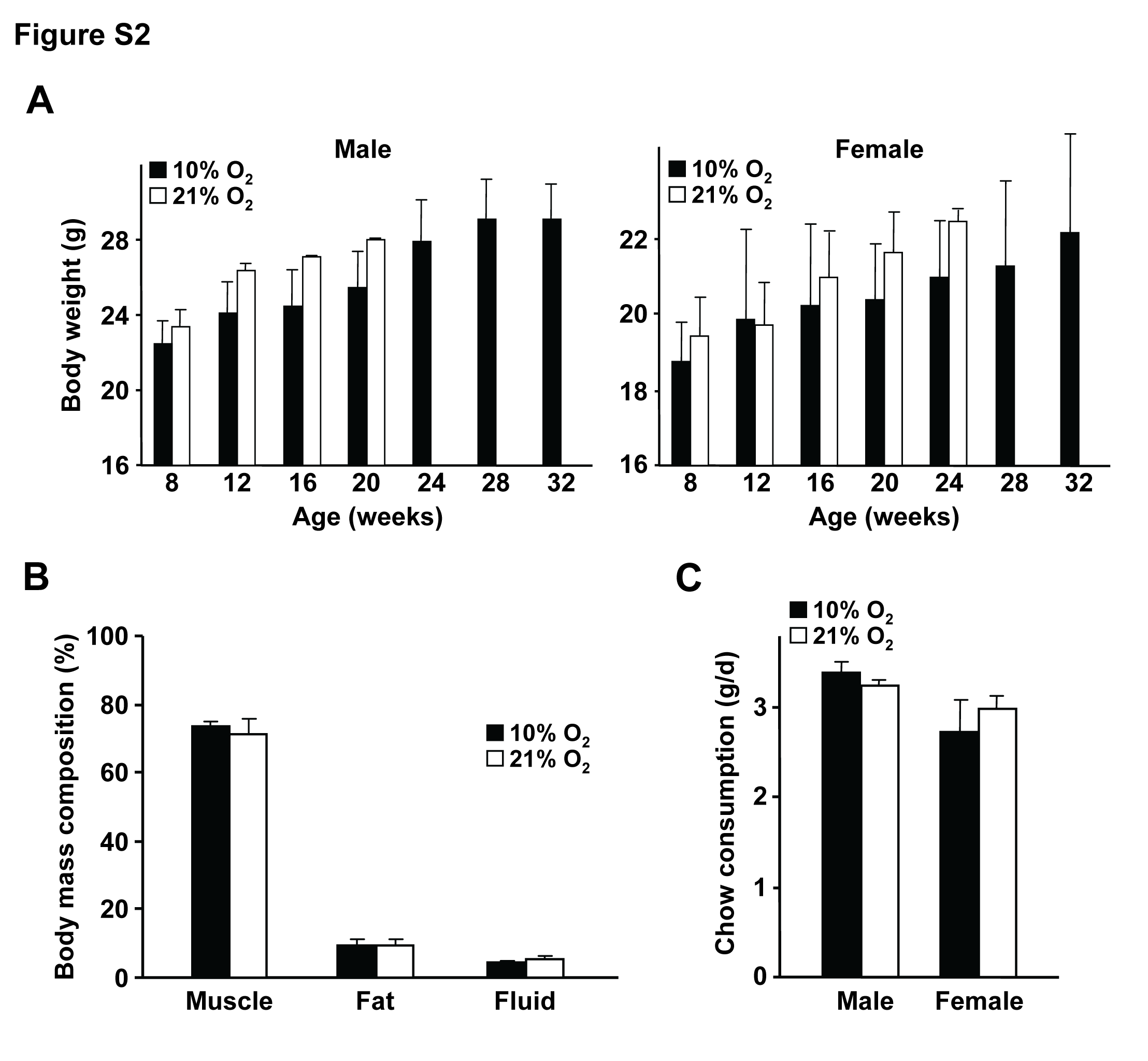

Supplement: Figure S2 — No significant difference in body weight, body mass composition or food intake in the 10% versus 21% oxygen condition. A. Body weights of male and female p53−/− mice in 10% oxygen versus 21% oxygen are shown over time. Mice with evidence of tumor were excluded. Note that the body weights of the 10% oxygen group span a longer period due to longer tumor-free survival time (n = 4 to 17 in each group). B. Body mass composition (% muscle, fat or free fluid of total body weight) was measured in non-anesthetized mice that were housed in 10% or 21% oxygen for up to 16 wk. Data are shown as mean ± SEM, with n = 3. C. Average food intake per day (g/d) by each mouse measured over a 4 wk period. Data are shown as mean ± SEM, with n = 5. (TIF) [file pone.0019785.s002.tif]

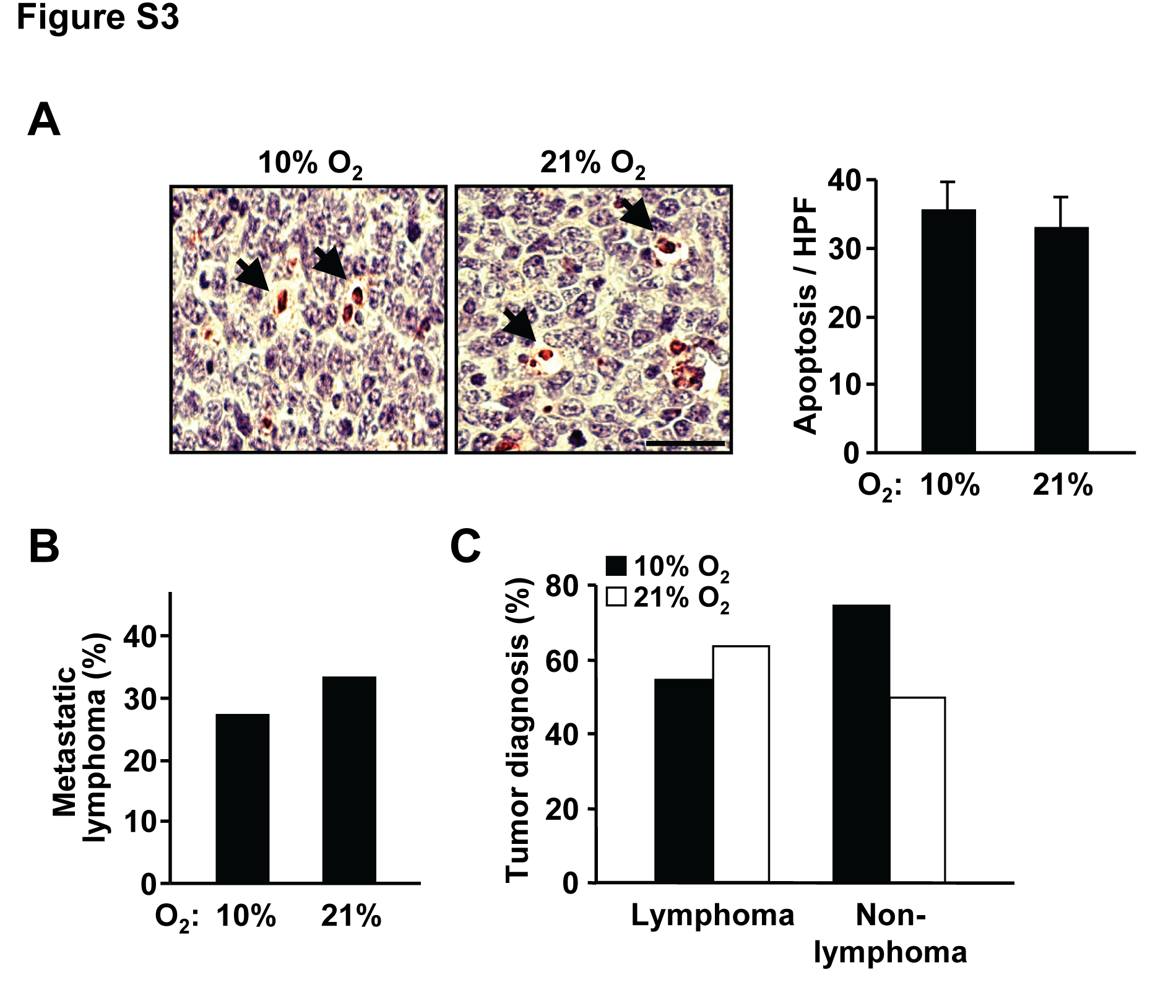

Supplement: Figure S3 — Lymphoma characteristics of p53−/− mice in 10% versus 21% ambient oxygen. A. Representative images of TUNEL stained thymic lymphoma show no significant differences in apoptosis between 10% and 21% oxygen conditions. The arrowheads indicate positively stained cells. Right panel shows the quantification of apoptotic nuclei per high power field in 5 to 10 separate regions. Data are shown as mean ± SEM, with n = 3. Scale bar, 20 µm (originally 40× magnification). B. No significant difference in the fraction of mice with metastatic lymphoma as determined by necropsy (10% oxygen, n = 20; 21% oxygen, n = 14). C. There is a trend of increased incidence of non-lymphoma tumors in 10% compared to 21% ambient oxygen condition (10% oxygen, n = 20; 21% oxygen, n = 14). (TIF) [file pone.0019785.s003.tif]

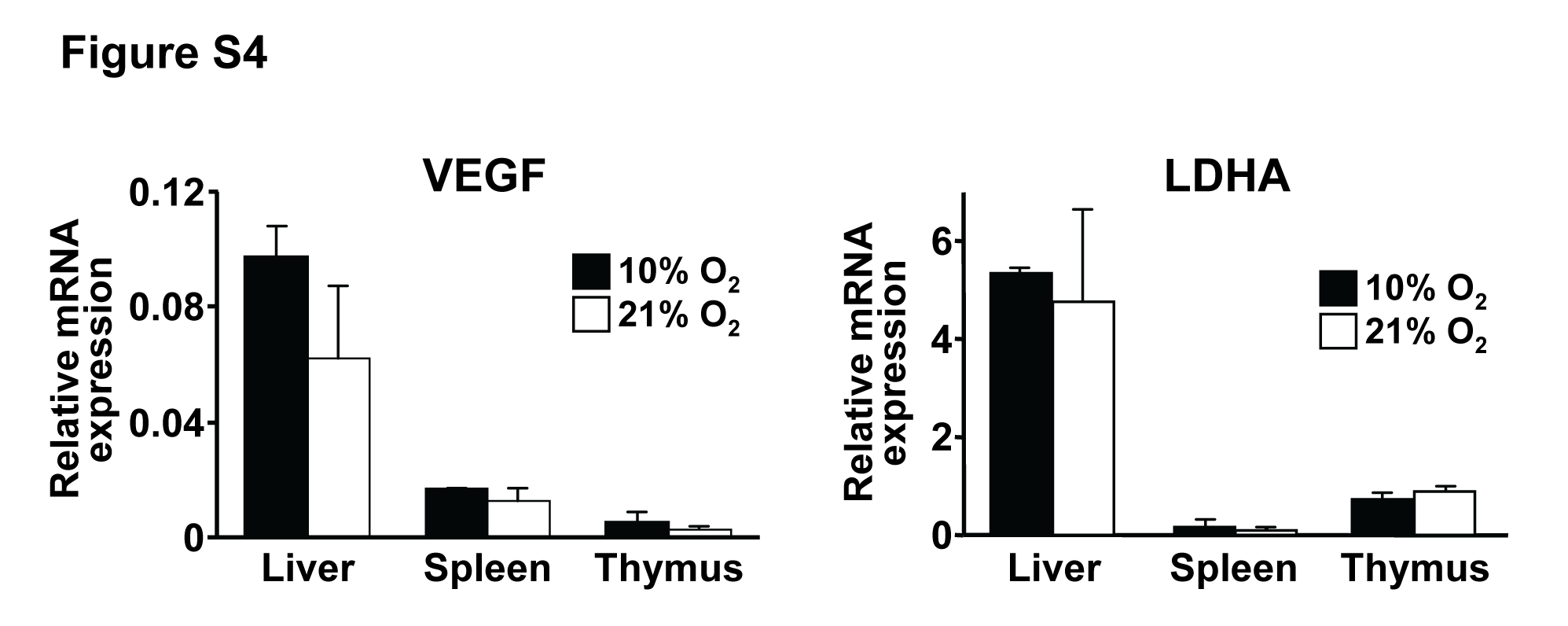

Supplement: Figure S4 — Expression levels of HIF1-α target genes are not significantly increased in 10% versus 21% oxygen. The relative levels of VEGF and LDHA mRNA as markers of HIF1-α activity were measured in liver, spleen and thymus of p53−/− mice. Mice were acclimated to 10% oxygen for at least 2 to 4 wk prior to tissue harvest. Data are shown as mean ± SEM, with n = 3. (TIF) [file pone.0019785.s004.tif]
